# Assessing the Protein Quality, In Vitro Intestinal Iron Absorption and Human Faecal Microbiota Impacts of Plant-Based Mince

**DOI:** 10.3390/nu16142339

**Published:** 2024-07-19

**Authors:** Damien P. Belobrajdic, Simone Osborne, Michael Conlon, Henri Brook, Rama Addepalli, Beverly S. Muhlhausler

**Affiliations:** 1 Health and Biosecurity, Commonwealth Scientific and Industrial Research Organisation, Adelaide, SA 5000, Australia; damien.belobrajdic@csiro.au (D.P.B.); michael.conlon@csiro.au (M.C.); henri.brook@csiro.au (H.B.); 2 College of Medicine and Public Health, Health Flinders University, Bedford Park, SA 5042, Australia; 3 Agriculture and Food, Commonwealth Scientific and Industrial Research Organisation, Brisbane, QLD 4067, Australia; simone.osborne@csiro.au (S.O.); rama.addepalli@csiro.au (R.A.); 4 School of Agriculture, Food and Wine, The University of Adelaide, Adelaide, SA 5005, Australia; 5 South Australian Health and Medical Research Institute, Adelaide, SA 5001, Australia

**Keywords:** plant-based, meat, nutrition, protein quality, fibre, iron, digestibility, intestinal absorption, microbiota

## Abstract

The nutritional quality of plant-based meat analogues compared to traditional meat products has been questioned in recent commentary, particularly in relation to protein quality and micronutrient bioavailability. However, the attributes of specific products within this category are unclear. We therefore undertook a comprehensive assessment of the compositional and functional attributes of v2food^®^ (Sydney, Australia) plant-based mince, including an assessment of the effects of reformulation, including the addition of amino acids, ascorbic acid, and different forms of elemental iron. The protein digestibility and protein quality of v2food^®^ plant-based mince were comparable to beef mince in the standardized INFOGEST system, and favourable effects on microbiota composition and short-chain fatty acid (SCFA) production were demonstrated in an in vitro digestion system. The use of ferrous sulphate as an iron source improved in vitro intestinal iron absorption by ~50% in comparison to other forms of iron (*p* < 0.05), although levels were ~3-fold lower than beef mince, even in the presence of ascorbic acid. In conclusion, the current study identified some favourable nutritional attributes of plant-based v2food^®^ mince, specifically microbiota and SCFA changes, as well as other areas where further reformulation could be considered to further enhance the bioavailability of key nutrients. Further studies to assess the effect of plant-based meat analogues on health measures in vivo will be important to improve knowledge in this area.

## 1. Introduction

The production and consumption of plant-based red meat substitutes have evolved in recent years to address concerns regarding sustainability, health, and environmental impacts of red meat consumption [1]. An increasing number of global companies are producing plant-based meat analogues and striving to create products that look and taste like animal meat and can be cooked in the same ways [1]. Despite advances in the look, taste and mouthfeel of plant-based meat analogues, the nutritional composition of most plant-based meat alternatives remains markedly different to animal meat, although some plant-based meat attributes, particularly the higher fibre content, may confer additional health benefits, including greater satiating effects [1,2].

Australians struggle to meet the recommended daily intakes for dietary fibre needed for optimal gut health [3]. In addition, Australians predominantly consume wheat fibre that assists with digesta bulking but is poorly fermented by our gut microbes [4]. Other fibres, such as fructans, beta-glucans and resistant starches, and foods which contain high levels of these, including specific wholegrains, legumes, and vegetables, are well recognized for promoting microbial fermentation, the production of bioactive metabolites, such as short-chain fatty acids (SCFAs), and subsequent functional benefits [4,5]. It is also important to consider the role the food matrix has in the microbiota response. A previous clinical study provided evidence that consuming plant-based meat was associated with some shifts in microbiota composition; however, limitations in the study design make it difficult to draw clear conclusions [6]. Therefore, it is important to establish whether the fibre provided by plant-based meat analogues can have a beneficial impact on microbiota composition and metabolite production.

Recent findings have also demonstrated the benefits of dietary fibre on metabolism and obesity [7]. For example, we previously demonstrated that a pasta bolognaise meal prepared with v2food^®^ plant-based mince was more satiating on a single meal occasion compared to an equivalent pasta meal prepared with beef mince [2], suggesting that plant-based meat alternatives may promote appetite control in the longer-term. The bioavailability and functionality of other macro- and micronutrients from plant-based meat analogues, compared to animal meat products, have only been investigated recently and remain largely unexplored.

Protein is one of the main macronutrients in both animal meats and plant-based meat analogues. Most plant-based meat products are formulated to achieve a total protein content equivalent to animal meats [1]. However, the amount of protein in a food is not the same as the protein quality, which is determined by protein digestibility and amino acid composition. While it is possible to obtain the full complement of essential amino acids from vegetarian, and even vegan, diets [8], this typically requires the inclusion of a variety of plant sources and, therefore, greater attention to the protein composition and content of different plant-based products when compared to diets that include animal protein. Plant proteins are also generally less digestible than animal protein as many plant-based protein sources contain anti-nutrients that can inhibit protein digestion and absorption [9,10]. Subsequently, it has been suggested that the protein quality of plant-based meat analogues is likely to be inferior to traditional meats, prompting calls for companies to provide information about the protein quality of plant-based meat alternatives. However, it is important to note that different plant proteins have different amino acid profiles. For example, soy protein is considered a complete protein, while other plant sources, such as pea protein, are incomplete protein sources that lack at least one essential amino acid [9,11]. The bioavailability of amino acids following digestion of plant protein can also be impacted by different anti-oxidant profiles and the food matrix [12]. Despite these limitations, consuming plant-based diets that include a variety of plant protein sources is generally considered sufficient to achieve an adequate amino acid intake [13].

Red meat is a major source of heme iron in human diets. While iron is present in several plant products, most notably green leafy vegetables such as spinach and kale, it is generally present at lower levels than in animal products [9,14]. The non-heme iron form present in plant sources is generally much less bioavailable than heme iron; however, it is important to note that some forms of non-heme iron, in particular the ferritin form, have a higher bioavailability than others [9,14]. In addition, anti-nutrients are present in most legumes used as base ingredients in plant-based meat products, including soy, further inhibiting iron absorption [12]. Consequently, the effect of consuming plant-based products on circulating iron levels is unclear, even though many plant-based meat alternatives are formulated with the same elemental iron concentrations as those found in equivalent animal products [9,14]. Therefore, there is a need to understand whether recipe modifications, such as the use of different forms of non-heme iron or the addition of ascorbic acid (a well-established enhancer of iron absorption) [15], could potentially increase iron absorption from these products.

The aim of the current study was to undertake a comprehensive analysis of the compositional and functional attributes of a plant-based mince produced by v2food^®^ and to determine whether reformulation of v2food^®^ plant-based mince could improve its protein quality and intestinal iron absorption.

## 2. Materials and Methods

### 2.1. Protein Quality

#### 2.1.1. Samples

Protein quality was assessed in the current standard v2food^®^ mince formulation and modified formulations that contained either (i) 0.39% methionine, (ii) 0.39% methionine and 0.15% cystine or (iii) 0.39% methionine and 0.15% N-acetyl-cystine. All plant-based mince formulations were provided by v2food^®^. Beef mince (4-star lean beef mince) was purchased from a supermarket.

#### 2.1.2. Sample Preparation

Raw samples of all mince products were cooked prior to analysis using an electric frying pan set to medium heat and minimal oil to prevent sticking. The preheated cooking surface was 120 °C at the end of the mince cooking time as measured by an infrared digital thermometer (NDI Tools, Cheltenham, Victoria, Australia). Beef mince and the v2food^®^ mince were formed into patties of 110 g. v2food^®^ mince was cooked in accordance with the instructions on the v2food^®^ website [16] such that samples were cooked for 4 min each side, then transferred to a plate with paper towel to aid the removal of excess oil. Cooked samples were subjected to a simulated chewing procedure prior to digestion, which involved performing 10 chops with a Zyliss ‘easy chop’ food chopper. A portion of the cooked sample was assessed as-is using the INFOGEST methodology, and another portion underwent freeze-drying and defatting procedures prior to amino acid determination.

### 2.2. Amino Acid Analysis

Amino acid content (excluding tryptophan and cysteine) was determined by acid hydrolysis, followed by detection by liquid chromatography–mass spectrometry. In brief, samples were hydrolysed in 1 mL of 6 M hydrochloric acid containing phenol and sodium thioglycolate, with 2-aminoisobutyric acid as an internal standard. Tubes were flushed with nitrogen and placed in a heat block at 110 °C for 18 h. Upon completion of the hydrolysis, samples were cooled, diluted with purified water, and centrifuged at 3000× *g* for 10 min. An aliquot of supernatant was diluted in acetonitrile and filtered using a 0.2 µm syringe filter prior to analysis. Detection of underivatized amino acids in the hydrolysates was performed by liquid chromatography–mass spectrometry, using a Shimadzu LCMS-9030.

Under the acid hydrolysis conditions used in this method, asparagine is hydrolysed to aspartic acid and glutamine to glutamic acid; therefore, these acids are reported as the sum of the respective components.

#### 2.2.1. Cysteine

Cysteine content was then determined by oxidation with performic acid, followed by acid hydrolysis and detection by liquid chromatography–mass spectrometry. Briefly, 0.5 mL of performic acid solution containing phenol was added to samples. The tubes were then sealed and incubated overnight (16 h) at 0 °C. The excess oxidant was decomposed using sodium metabisulfite and the contents of the tubes were evaporated under a flow of nitrogen at 40 °C until dryness. Then, 1 mL of 6 M hydrochloric acid containing phenol was added and samples were hydrolysed in a heat block at 110 °C for 24 h. The samples were then cooled, diluted with purified water, and centrifuged at 3000× *g* for 10 min. An aliquot of the supernatant was diluted in acetonitrile, spiked with internal standard (2-aminoisbutyric acid), and filtered using a syringe filter (0.2 µm).

#### 2.2.2. Tryptophan

Tryptophan content was determined by base hydrolysis, with detection by liquid chromatography–mass spectrometry. Briefly, samples were hydrolysed in 3 mL of 4 M lithium hydroxide containing ascorbic acid. Sample tubes were then sealed and placed in a heat block at 110 °C for 16 h. Following hydrolysis, the samples were cooled and neutralized with hydrochloric acid. An aliquot was filtered through a syringe filter (0.2 µm), diluted with purified water, and spiked with internal standard (Tryptophan-d5, Toronto Research Chemicals, Toronto, ON, Canada) for quantification.

#### 2.2.3. In Vitro Protein Digestibility

All cooked mince products were digested in triplicate using the INFOGEST 2.0 protocol [17] with adjustments for pancreatin solubilization and application of a modified workflow, as previously described [18]. Each mince sample, providing 40 mg of protein, was combined with 250 mg of a protein-free biscuit to form a test meal. Samples were digested using simulated oral, gastric, and intestinal digestion containing appropriate gastrointestinal tract enzymes, bile salts, pH values, incubation times, and temperature (37 °C). Digested samples were precipitated in 80% methanol with supernatant and pellet portions separated for amino acid analyses. The reference Digestible Indespensable Amino Acid (DIAA) values used to calculate the DIAA scores (DIAASs) were determined as described in the FAO report on dietary protein quality evaluation in human nutrition [19].

### 2.3. Fibre Study

#### 2.3.1. Samples

The assessment of fibre composition was conducted on 4 separate batches of v2food^®^ mince purchased from a supermarket, and these same batches were used as a substrate for in vitro fermentation.

#### 2.3.2. Fibre Composition

Total dietary fibre, soluble fibre, and insoluble fibre composition was determined in raw mince using AOAC method 991.43.

#### 2.3.3. In Vitro Digestion and Fermentation

Raw v2food^®^ mince (a mixture of the 4 batches added in equal amounts) was subjected to an in vitro process which simulates upper gastrointestinal tract digestion, as described previously [20]. Briefly, the mince was digested in a 0.02 M HCL solution containing 1 mg/mL pepsin for 30 min at 37 °C to simulate gastric digestion. To initiate upper intestinal digestion, the gastric digesta was adjusted to pH 6.0 with 0.2 M sodium acetate, before 25 units/mL of amyloglucosidase and one capsule of Creon 10,000 pancreatic extract in 0.2 M sodium acetate was added to digesta and incubated for 16 h at 37 °C. The non-digested residue was precipitated with 80% ethanol and centrifugation. The pellet was washed with acetone and then air-dried. The resulting non-digestible residue was used as a substrate for in vitro fermentation.

A modification of an in vitro anaerobic batch fermentation method, which uses a human faecal inoculum, was conducted to simulate fermentation that occurs in the human gut. The method, including preparation of donor stool and information on buffers and medium, was performed as described previously [20]. Briefly, fresh stool samples were obtained from 2 healthy adult individuals, diluted in phosphate-buffered saline, homogenized, and refrigerated (at 4 °C). The faecal slurry was then removed from refrigeration, diluted to 10% (*w*/*v*) in fermentation medium with 10 mL added to separate fermentation test tubes. Then, 100 mg of digested v2food^®^ mince, cellulose (poorly fermentable substrate control) or inulin (fermentable substrate control) was then added to each fermentation tube. Blank tubes that contained all components, including diluted faecal slurry but no added substrate, were also included as a negative control. All treatments were fermented in triplicate (3 fermentation tubes each). The tubes were sealed, incubated at 37 °C under anaerobic conditions, and mixed using continuous orbital shaking for 24 h. Aliquots of the ferments were stored at −80 °C.

### 2.4. SCFA Analysis

The levels of SCFAs in medium following 24 h of fermentation were determined by an established gas chromatography analysis method (described elsewhere [21,22]).

### 2.5. Microbiota Analysis

Extraction of microbial DNA from ferment samples and subsequent sequencing of the 16S rRNA region was carried out by the Australian Genome Research Facility (AGRF). Assessment of microbial population changes was conducted by PCR amplification of the 16S rRNA region of extracted DNA and sequencing of the amplified DNA; 300 bp sequencing of the V1–V3 region of the 16S rRNA region was performed using an Illumina MiSeq. Paired-end reads were assembled by aligning the forward and reverse reads using PEAR (version 0.9.5). Primers were identified and trimmed, and trimmed sequences were processed using Quantitative Insights into Microbial Ecology (QIIME 1.8.4) [23] and USEARCH (version 8.0.1623) [24] software, using the UPARSE [25] algorithm for generation of clusters. Using search tools, sequences were quality-filtered; full-length duplicate sequences were removed and sorted by abundance. Single or unique reads in the data set were discarded. Sequences were then clustered and chimera filtered using the “rdp gold” database as reference. To obtain the number of reads in each Operational Taxonomic Unit (OTU), reads were mapped back to OTUs with a minimum identity of 97%. Taxonomy was assigned using the QIIME 1.8.4 microbiota bioinformatics platform.

### 2.6. Intestinal Iron Absorption In Vitro

#### 2.6.1. Sample Formulations

Intestinal absorption of iron in v2food^®^ mince was assessed in formulations containing different forms of elemental iron and different iron levels with or without inclusion of ascorbic acid.

Six commercial iron sources were investigated (Ferrous fumarate, Ferric EDTA, Ferrous sulphate, Ferric pyrophosphate, Ferrous bisglycinate, and ferrous bisglycinate packaged with citric acid, maltodextrin, and silica) at three different inclusion levels: (i) current inclusion level in v2food^®^ mince—2 mg elemental iron added to 100 g uncooked wet weight, total iron content 2.8 mg/100 g; (ii) maximum claimable iron levels as defined by FSANZ—2.7 mg elemental iron added per 100 g uncooked wet weight, total iron content 3.5 mg/100 g; or (iii) double the current commercial iron levels—4.0 mg elemental iron added per 100 g uncooked wet weight, total iron content 4.8 mg/100 g mince.

The three different inclusion levels of elemental iron were investigated with and without ascorbic acid. Each iron source was added to the v2food^®^ mince to deliver the same levels of elemental iron. To assess the impact of ascorbic acid, intestinal iron absorption of all formulations was determined both in the absence of ascorbic acid and in the presence of ascorbic acid at 2:1 and 4:1 molar ratios of ascorbic acid/iron. All v2food^®^ mince combinations were compared to lean beef mince (i.e., 10% fat), which typically contains 2 mg iron/100 g mince (wet weight).

#### 2.6.2. Sample Preparation and Cooking

All v2food^®^ mince combinations were prepared in accordance with the protocol provided by v2food^®^. Briefly, base ingredients (textured vegetable protein, colour and flavour blends, water and canola oil) and test ingredients (elemental iron and ascorbic acid at the required levels) were mixed by hand at room temperature. The mixture was then cooled (to <2 °C) before the addition of coconut fat. The resulting mixture was formed into 125 g patties of 9–10 cm diameter. Beef mince patties of equal size and weight were formed and all patties chilled or frozen until ready to cook. Frozen patties were thawed in a fridge (4 °C) for 48 h before cooking and removed from the fridge 1 h before cooking to allow for a pre-cooking temperature of >10 °C. Patties were cooked on a non-stick grill preheated to 180 °C for 3–5 min or until they reached an internal temperature of 74 °C.

#### 2.6.3. In Vitro Digestion

Prior to digestion, all project samples were freeze-dried to remove moisture. To simulate human oral and gastrointestinal digestion in vitro, the INFOGEST static in vitro digestion method was used, as described above. Samples of digest were collected at 60 min after the start of the intestinal digestion phase, filtered using a 10 kDa filter, and stored at −20 °C.

#### 2.6.4. In Vitro Assessment of Intestinal Iron Absorption

A transwell intestinal model, comprising human Caco-2 (enterocytes) and HT29-MTX-E12 (goblet-like) cells grown on a semi-permeable membrane, was used to assess intestinal absorption of iron from digesta of all test formulations and beef mince, as described in detail previously [26]. Briefly, Caco-2 (3.6 × 10^4^ cells) and HT29-MTX-E12 (4 × 10^3^ cells) cells were added to the apical chamber of 0.33 cm^2^ transwells containing 0.6 mL of cell-free growth media. After 21 d, during which growth media was changed every 2–3 days, transepithelial electric resistance (or TEER) was measured using a Millicell voltameter to confirm integrity of the cell layer and suitability of the cell system for subsequent experiments.

Following preparation of an intact intestinal cell barrier, samples of digesta of all test formulations and beef mince were prepared in Hank’s Buffered Saline Solution (HBSS) at a 1:6 dilution (which was confirmed to be non-toxic to cells in cell viability studies). Prior to the addition of the digesta, the growth media in the transwell system was replaced with HBSS for 2 h at 37 °C to deplete cells of fetal bovine serum. The HBSS was then replaced with diluted digesta and incubated at 37 °C for 2 h. The diluted digesta was then removed from the cells, replaced with HBSS, and incubated for 16–18 h at 37 °C. At the end of the incubation period, cells on the apical side of the transwell were washed with PBS before being removed from the transwell membranes through the addition of trypsin. Cells were collected by 5 min of centrifugation, and ferritin concentrations in the cells were assessed using the Abcam human ferritin ELISA kit (ab200018), according to the manufacturer’s instructions.

### 2.7. Statistical Analyses

Total protein digestibility, fibre content, and SCFA production following digestion and fermentation were compared between beef mince and the different v2food^®^ mince preparations, using a one-way ANOVA followed by Tukey’s post hoc test to assess significant differences between individual products and formulations. Effects on different bacterial species at both the phylum and genus level were similarly determined.

For the intestinal iron absorption studies, results from the ferritin assays were analyzed for significant differences using either unpaired *t*-tests or one-way ANOVA and Tukey’s post hoc tests. To control for any cell variations and differences in ferritin detection (by ELISA) across experiments, all findings are expressed as percentages relative to the ferrous sulphate control.

All data were analyzed using Graphpad Prism (version 9) and *p* < 0·05 indicated significance for all analyses.

## 3. Results

### 3.1. Protein Quality

#### Amino Acid Composition

The total amino acid content was similar between beef mince and all v2food^®^ formulations (beef mince, 26.05 g/100 g; v2 control, 18.65 g/100 g; v2 formulation 1, 19.33 g/100 g; v2 formulation 2; 19.48 g/100 g; v2 formulation 3, 20.03 g/100 g). The sulphur amino acid content (SAA, sum of methionine and cysteine) was higher in v2food^®^ mince than in the beef mince (beef mince, 0.82 g/100 g; v2food^®^ mince, 1.35 g/100 g), mostly due to the higher cysteine content in the v2food^®^ mince (Table 1). However, the levels of all other amino acids were lower in the v2food^®^ mince compared to the beef mince (Table 1). The branched chain amino acid content (BCAA, sum of leucine, isoleucine and valine) of v2food^®^ mince was also lower than beef mince (beef mince, 4.75 g/100 g; v2food^®^ mince, 3.05 g/100 g).

Adding methionine and/or cysteine to the control v2food^®^ mince product increased the total SAA content, as expected (beef mince, 0.82 g/100 g; v2food control, 1.35 g/100 g; v2food formulation 1, 1.64 g/100 g; v2 formulation 2; 1.55 g/100 g; v2 formulation 3, 1.67 g/100 g).

### 3.2. Protein Digestibility

**Figure 1:**
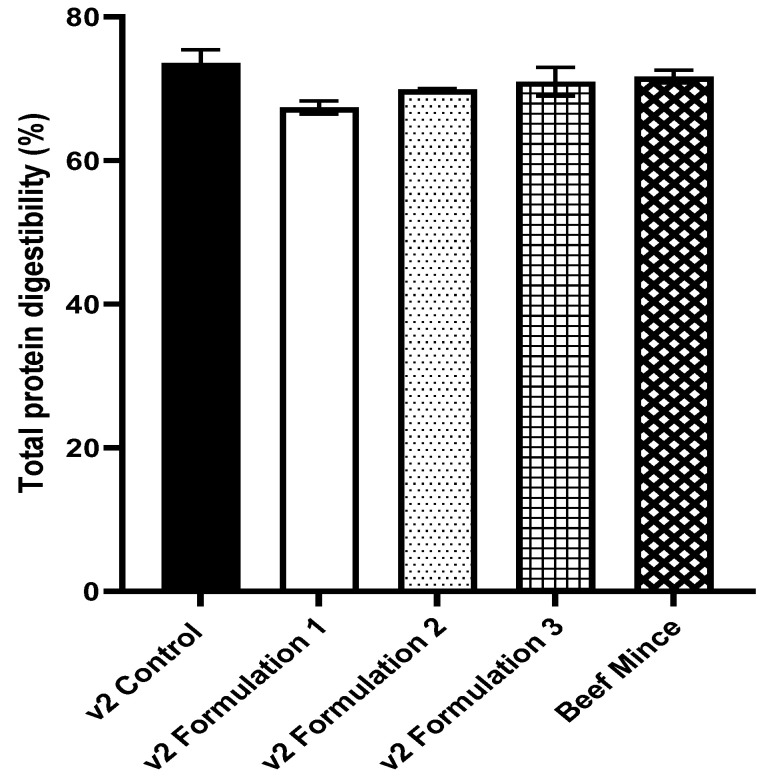
Total protein digestibility (%) as determined by INFOGEST (mean ± SEM). There were no significant differences in total protein digestibility between groups (one-way ANOVA, *p* = 0.28).

The total protein digestibility of original v2food^®^ mince (73.6%) was similar to beef mince (71.7%) and all three reformulated v2food^®^ mince types (Figure 1).

The protein digestibility of individual amino acids also varied for the different types of mince. v2food^®^ mince had higher digestibility of Phe and aromatic amino acids (AAAs) compared to beef mince (Table 2). For sulphur-containing amino acids (SAAs), Met digestibility was higher for v2food^®^ mince compared to beef mince, while Cys digestibility was lower (Table 2). The overall digestibility of SAAs was lower for v2food^®^ mince compared to beef mince (beef mince, 64.6 ± 1.7%; v2food^®^ mince, 51.4 ± 2.0%; *p* = 0.011; Table 2), mostly due to the higher relative abundance of Met compared to Cys in the v2food^®^ mince.

In vitro DIAASs, based on older child, adolescent, and adult reference values, are presented in Table 3. The in vitro DIAAS for v2food^®^ mince was similar to beef mince (beef mince, 69.6; v2food^®^ mince, 70.9%, *p* = 0.816; Table 3). The main limiting amino acids for v2food^®^ mince products were tryptophan (70.9%) and valine (73.2%). For beef mince, the main limiting amino acid was valine (69.6%) (Table 3). When in vitro DIAASs were determined using the older child reference values, tryptophan was the limiting amino acid for all mince products tested (Table 3).

When DIAA reference ratios were calculated, which take into account the amount of each amino acid present per gram of protein in each sample type, the amount of available SAAs in the v2food^®^ samples was higher than in beef mince products (*p* < 0.01). When the DIAA was compared to the adult reference values to obtain an in vitro digestible amino acid score (DIAAS), the reformulations of v2food^®^ mince all gave scores of over 2.0, which was higher than the v2food^®^ control (1.62) and beef mince (0.88) (Table 3).

### 3.3. Fibre Composition and Effects on Microbiota

#### 3.3.1. Fibre Content and Composition

The v2food^®^ mince product had a total fibre content of 6.7 ± 0.1% wet weight, most of which was insoluble fibre (5.0 ± 0.1% wet weight of insoluble fibre vs. 1.8 ± 0.1% wet weight of soluble fibre).

#### 3.3.2. Effects on SCFA Production

Following in vitro digestion and 24 h of fermentation in a system designed to replicate the human digestive tract, significant increases were observed in the concentrations of SCFAs, acetic acid, butyric acid, and propionic acid compared to the no-substrate control (Control) and cellulose-negative control (Table 4). Increases in propionic acid and butyric acid following fermentation were also higher for the digested v2food^®^ product than for inulin (positive control). Inulin and v2food^®^ fermentation also reduced pH compared to Control.

#### 3.3.3. Effects on Microbiota Composition

In vitro fermentation of digested v2food^®^ mince was associated with significant shifts in stool-derived bacteria populations at both the phylum (Table 5) and genus (Table 6) levels when compared to controls.

Across all treatments, bacteria of the Bacillota and Bacteroidota phyla were predominant, representing over 90% of the population, with the Bacillota at 84 to 89% abundance for the control and cellulose treatments, respectively. Actinomycetota and Pseudomonadota each made up approximately 1–3% of the population, whereas bacteria belonging to Lentisphaerota, Synergistota, Mycoplasmatota, and Verrucomicrobiota were present at less than 0.1%. Addition of the v2food^®^ mince digesta was associated with increases in the abundance of Actinobacteria and decreases in the abundance of Lentisphaerae, Mycoplasmatota, and Verrucomicrobiota relative to the control.

A total of 59 distinct genera were identified by sequencing. Fermentation with the digested v2food^®^ mince significantly impacted the abundance of 19 genera compared to the control, and 13 genera relative to both the control and cellulose treatments (Alistipes, Anaerostipes, Clostridium, Collinsella, Eggerthella, Faecalibacterium, Haemophilus, O2DO6, Oscillospira, Prevotella, SMB53, Streptococcus, and Subdoligranulum) (Table 6).

### 3.4. Intestinal Iron Absorption In Vitro

#### Effect of Elemental Iron Source and Ascorbic Acid

Beef mince stimulated ferritin production approximately three times more than any v2food^®^ plant-based meat formulation (Table 7).

v2food^®^ mince formulations containing ferrous sulphate stimulated ferritin production significantly more than ferric pyrophosphate at all inclusion levels (Table 7).

Inclusion of ascorbic acid, at either 1:2 or 1:4 iron/ascorbic acid molar ratios, had small but significant effects on ferritin production which were dependent on both the form of iron and the iron level. At the lowest iron level (2 mg/100 g), equivalent to levels in current, commercially available v2food^®^ mince, inclusion of 1:2 and 1:4: iron/ascorbic acid molar ratios significantly increased cellular ferritin production in response to ferric pyrophosphate, but not other forms of iron. At an iron inclusion level of 2.7 g/100 g (maximum iron level allowed by FSANZ), the addition of ascorbic acid at the 1:2 ratio, but not the 1:4 ratio, significantly increased intestinal iron absorption from the ferrous fumarate formulation to levels equivalent to those for ferrous sulphate, but inclusion of ascorbic acid at either molar ratio did not significantly improve cellular ferritin production in response to any other iron sources. At the highest iron level of 4 mg/100 g (double the current commercial inclusion iron level), addition of a 1:2 iron/ascorbic acid molar ratio significantly increased ferritin production in v2food^®^ mince prepared with ferrous bisglycinate packaged with citric acid. However, addition of a 1:4 molar ratio of ascorbic acid at the highest iron level did not significantly improve cellular ferritin production in response to any form of iron (Table 7).

## 4. Discussion

The studies described here were designed to investigate the compositional attributes and functional properties of v2food^®^ plant-based mince and gain a comprehensive understanding of its protein quality (amino acid composition and digestibility), fibre composition and effects on the gut microbiota, and intestinal iron absorption. Importantly, these attributes were compared directly to beef mince. We found that protein digestibility and overall protein quality (as assessed by in vitro DIAAS) of the v2food^®^ products were comparable to beef mince. As expected, the fibre content of v2food^®^ plant-based mince was considerably higher than that reported for traditional meat products, and the in vitro fermentation studies indicated that the v2food^®^ mince altered microbial populations and increased SCFA production, indicative of favourable gut health effects [27]. Intestinal iron absorption was significantly lower in the v2food^®^ products compared to lean beef mince, and even though inclusion of ferrous sulphate as the elemental iron source improved in vitro intestinal iron absorption from reformulated v2food^®^ plant-based mince, levels remained below intestinal iron absorption from beef mince.

In the current study, protein quality assessments included amino acid composition and in vitro digestibility and demonstrated that v2food^®^ mince protein quality is comparable to beef mince. This contrasts with a previous study where lower protein quality was observed in plant-based meat products compared to traditional meat products [18]. However, our findings are similar to an in vivo study in pigs that published a DIAAS of 91 for Impossible plant-based products which are made from soy protein and reported similar protein quality to animal meat [28,29]. In this same study, a DIAAS of only 71 was reported for another product made from pea protein. Collectively, these results suggest considerable variations in protein quality across the major commercially available plant-based mince products and highlight the importance of providing consumers with access to information about the attributes of specific products. That the v2food^®^ plant-based mince had a higher protein digestibility than its base protein ingredient (i.e., soy) suggests that protein digestibility is enhanced by the processing methods used in the production of these plant-based products. A review by Shan and colleagues published in 2023 provides some insights into formulations and processing techniques that can influence the protein digestibility of plant-based meat analogues [30]; however, further studies are needed to explore processing techniques that are likely to achieve optimal protein digestibility, as well as explore how different processing techniques impact other product attributes.

While the protein quality of v2food^®^ plant-based mince was similar to meat, the concentrations of most amino acids, including essential amino acids, were lower in all formulations of the v2food^®^ plant-based products. Notably, methionine and glycine concentrations were lower in v2food^®^ plant-based mince and were consistent with low levels of these amino acids in the base ingredient (i.e., soy) [31,32]. While these amino acid levels could be increased by reformulation, it is also important to note that these amino acids are present in a wide range of other foods, including eggs, rice, and nuts, so the risk of deficiency is unlikely when plant-based meat products are consumed as part of a nutritionally balanced diet [13]. In addition, despite methionine intake being lower in vegetarians compared to individuals who eat meat and fish, previous research has shown that blood concentrations of methionine are higher in vegetarians, suggesting that intake, blood concentration, and possible physiological effects are not necessarily directly related [33].

The multitude of health benefits of consuming higher quantities of plant-based foods has been largely attributed to their high content of dietary fibre [12,34]. In this study, the dietary fibre content of v2food^®^ mince was found to be 6.7% wet weight, so a standard serve (125 g) of v2food^®^ mince would be expected to provide ~8.4g of fibre, representing ~34 and 28% of the Australian recommendation for total dietary fibre intakes in women and men, respectively [3]. In addition, v2food^®^ mince contains both soluble and insoluble fibre, both of which play an important role in maintaining gut and metabolic health [4,5]. Importantly, in vitro digestion and fermentation analyses demonstrated that this type of fibre modulated the microbiota composition and SCFA production. Increased SCFA production is a well-established marker of favourable shifts in the composition and activity of the gut microbiota [27]. A particularly interesting finding was that production of two SCFAs, propionic acid and butyric acid, was higher following fermentation of the v2food^®^ product, compared to inulin—a probiotic that is usually included as a positive control. While the specific functions of propionic acid are not well understood, butyric acid is the primary energy source for cells lining the colon that stimulates apoptosis of damaged cells in the gut, contributes to lowering pH, which has functions to suppress pathogenic bacteria and plays an important role in supporting immune system functionality [35,36].

The composition of the gut microbiota is now known to play a critical role in both gastrointestinal and overall health, and higher-fibre diets have previously been associated with beneficial shifts in microbial populations [5]. The v2food^®^ digesta in the fermentation system was associated with increases in the abundance of key butyric acid producers, including *Faecalibacterium*, consistent with the increased butyric acid production induced by v2food^®^ mince, and commensal bacteria, including *Prevotella* [37,38]. Similarly, v2food^®^ digesta was associated with a reduced abundance of bacterial genre that have been associated with inflammation, infection, and gut pathology, including *Haemophilus*, *Alistipes*, *Eggerthella*, *Streptococcus*, *Oscillospira*, and *Collinsella* [39,40,41,42,43]. While these findings support previously reported positive impacts of plant-based meat products on gut microbiota and gut health, it is not possible to directly extrapolate the findings from our in vitro system to the in vivo setting, and appropriately designed and powered human clinical trials are required to confirm these effects.

Absorption of plant-derived, non-heme iron has consistently been reported to be lower than that of heme iron from animal products [15], similar to the findings of the current study. Intestinal absorption of iron from v2food^®^ mince was ~3 times lower than intestinal iron absorption from beef mince in the in vitro system. However, it is important to note that many plant-based meats, including v2food^®^ mince, are formulated to achieve a total iron content aligned with the FSANZ requirements for a ‘source of iron’ claim, and that these fortified products provide a superior source of iron when compared to plant-based products without supplemental iron. Interestingly, a previous study reported that in vitro intestinal iron absorption from a soy-based burger did not differ significantly from beef [9]; however, this may have been overestimated, as the method used did not account for iron uptake by cells. In contrast, measuring ferritin, an intracellular protein that stores iron, as in the current study, replicates clinical practise and provides a direct measure of intestinal iron absorption in vitro [44].

The form of elemental iron also influenced intestinal iron absorption, with v2food^®^ mince formulations containing ferrous sulphate, increasing intestinal iron absorption ~50% more than all other forms, independent of the elemental iron level. These findings are consistent with previous studies that have reported increased absorption of iron from ferrous sulphate [45], but to the best of our knowledge, this is the first study to directly compare different iron sources in a plant-based meat matrix. Addition of ascorbic acid did not consistently improve intestinal iron absorption in our study, with the greatest improvements in intestinal iron absorption observed for ferric iron sources, consistent with the mechanism of action of ascorbic acid for reducing ferric iron into ferrous iron [15]. While the lack of any significant improvements in iron uptake by the addition of ascorbic acid is unexpected, it possible that the presence of other components in the v2food^®^ mince, in particular anti-nutrients including phytates, could have offset the benefits of ascorbic acid inclusion. Given the potential benefits of strategies to increase absorption of non-heme iron, particularly for population groups with higher iron requirements, in vivo studies to assess the impact of other reformulations are warranted.

Strengths of this study include the use of well-established and validated in vitro methods to assess both the composition and functional properties of v2food^®^ plant-based mince without the use of animal models. We tested a broad range of measures, which enabled us to obtain a comprehensive picture of the nutritional attributes of these plant-based products, and their potential health benefits. The use of in vitro models also provided the opportunity to investigate the potential benefits of reformulation on protein quality and iron absorption, to inform future studies. While this study had many strengths, there are also limitations. While the in vitro methods applied are well accepted in the field, no in vitro method can fully replicate the in vivo situation, and in vivo, clinical studies are required to confirm any health benefits associated with the consumption of these products. In the case of iron absorption studies, for example, it is difficult to fully account for the homeostatic mechanisms that control intestinal iron absorption and secretion in vivo in an experimental system, and this needs to be considered in the interpretation of the findings. Similarly, while the in vitro gut model provides insights into the potential role of v2food^®^ plant-based mince in producing favourable effects on gut health, the model does not fully account for the variability in microbiota composition and metabolism between individuals and requires in vivo confirmation.

## 5. Conclusions

In the current study, we have demonstrated that v2food^®^ plant-based mince has a protein digestibility and protein quality that is comparable to beef mince based on the standardized INFOGEST method. The v2food^®^ plant-based mince also provided levels of dietary fibre that stimulated gut microbiota composition and metabolic activity. While the use of ferrous sulphate as an iron source improved intestinal iron absorption in comparison to other forms of iron, iron absorption remained substantially lower than beef mince, even in the presence of ascorbic acid. The current study identified some favourable nutritional attributes of plant-based v2food^®^ mince, specifically microbiota and SCFA changes, as well as other areas where further reformulation could be considered to further enhance the bioavailability of key nutrients. Further studies to assess the effect of plant-based meat analogues on health measures in vivo will be important to improve knowledge in this area.

## Figures and Tables

**Table 1:** Amino acid concentrations in beef mince and v2food^®^ formulations (g/100 g).

	Ala	Thr	Phe	Leu	Ile	Met	Val	Pro	Tyr	Gly	Ser	Glx ^1^	Asx ^2^	His	Arg	Lys	Trp	Cys
Beef Mince	1.66	1.44	0.63	2.52	1.09	0.51	1.14	1.08	1.01	1.55	1.33	2.99	1.87	1.66	2.77	2.32	0.28	0.31
v2food^®^ Control	0.88	0.99	0.48	1.52	0.76	0.17	0.77	0.68	0.81	0.71	1.11	2.35	1.61	0.88	2.01	1.42	0.21	1.18
v2food^®^ Formulation 1	0.91	1.01	0.50	1.47	0.70	0.44	0.78	0.70	0.81	0.73	1.14	2.45	1.66	0.91	2.08	1.47	0.22	1.20
v2food^®^ Formulation 2	0.92	1.01	0.50	1.56	0.79	0.52	0.83	0.68	0.81	0.73	1.13	2.46	1.66	0.92	2.08	1.48	0.22	1.03
v2food^®^ Formulation 3	0.94	1.04	0.49	1.54	0.80	0.45	0.85	0.70	0.85	0.73	1.16	2.52	1.75	0.94	2.13	1.51	0.22	1.22

^1^ Glx, sum of glutamine and glutamic acid; ^2^ Asx, sum of asparagine and aspartic acid.

**Table 2:** Mean protein digestibility (%) of (**a**) beef mince vs. the v2food^®^ control mince and (**b**) the different v2food^®^ formulations ^1^.

	Ala	Thr	Phe	Leu	Ile	Met	Val	Pro	Tyr	Gly	Ser	Glx ^2^	Asx ^3^	His	Arg	Lys	Trp	Cys	SAAs ^4^	AAAs ^5^	Total ^6^
(**a**)																					
Beef Mince	80.4	68.2	68.5	72.0	68.9	69.7	63.6	72.9	63.8	76.1	68.5	73.7	66.5	79.4	78.2	69.6	52.8	55.4	64.6	65.8	71.7± 0.9
v2food^®^ Control	76.8	69.6	80.0	77.8	76.1	77.2	70.6	76.8	73.5	70.6	69.9	80.9	69.0	81.6	80.7	74.2	42.3	47.3	51.4	76.2	73.6 ± 1.8
*p*-value	0.061	0.483	0.001	0.171	0.221	0.045	0.021	0.313	0.010	0.302	0.531	0.060	0.245	0.774	0.456	0.206	0.042	0.678	0.011	0.002	0.341
(**b**)																					
v2food^®^ Control	76.8	69.6	80.0	77.8 ^b^	76.1 ^ab^	77.2 ^a^	70.6 ^b^	76.8	73.5	70.6	69.9	80.9 ^b^	69.0	81.6 ^b^	80.7 ^b^	74.2	42.3	47.3	51.4 ^a^	76.2	73.6 ± 1.8
v2food^®^ Form.1	73.3	63.9	76.2	72.7 ^a^	71.0 ^a^	87.6 ^b^	62.3 ^a^	69.6	68.2	61.3	61.6	71.6a	63.0	65.3 ^a^	71.6 ^ab^	65.3	40.0	48.0	61.0 ^b^	71.5	67.4 ± 0.9
v2food^®^ Form.2	76.3	66.6	82.5	79.3 ^b^	77.2 ^b^	92.0 ^b^	69.7 ^b^	75.1	72.8	63.4	61.9	72.6 ^ab^	64.5	65.5 ^a^	71.1 ^a^	67.3	49.6	45.9	62.2 ^b^	76.8	69.9 ± 0.1
v2food^®^ Form.3	76.2	66.3	80.1	78.5b	77.6 ^b^	90.1 ^b^	71.4 ^b^	71.4	71.3	65.2	63.1	74.7 ^ab^	66.6	69.3 ^ab^	73.7 ^ab^	70.1	48.0	52.5	63.1 ^b^	75.0	71.0 ± 2.0
*p*-value	0.061	0.483	0.001	0.171	0.221	0.045	0.021	0.313	0.010	0.302	0.531	0.060	0.245	0.774	0.456	0.206	0.042	0.678	0.011	0.002	0.341

^1^ Different superscripts denotes significant differences between groups at *p* < 0.05 (ANOVA and Tukey post hoc test); ^2^ Glx, sum of glutamine and glutamic acid; ^3^ Asx, sum of asparagine and aspartic acid; ^4^ SAAs, sulphur amino acids (sum of methionine and cysteine); ^5^ AAAs, aromatic amino acids (sum of phenylalanine and tyrosine); ^6^ Total, average protein digestibility of all amino acids.

**Table 3:** In vitro digestible indispensable amino acid (DIAA) reference ratio and digestible indispensable amino acid score (DIAAS) for an older child, adolescent, and adult, as measured by INFOGEST for (**a**) beef mince vs. the v2food^®^ control mince and (**b**) the different v2food^®^ formulations. Amino acids in brackets indicate limiting amino acid for that product.

(**a**)					
	**Beef Mince**		**v2food^®^ Mince**		***p*** **-Value**
DIAA reference ratio					
Threonine	1.51 ± 0.02		1.48 ± 0.04		0.477
Leucine	1.14 ± 0.05 ^b^		1.04 ± 0.02 ^a^		0.012
Isoleucine	0.96 ± 0.06		1.03 ± 0.02		0.335
Valine	0.70 ± 0.20		0.73 ± 0.01		0.167
Histidine	2.98 ± 0.10		2.78 ± 0.17		0.366
Lysine	1.29 ± 0.01		1.18 ± 0.05		0.099
Tryptophan	0.85 ± 0.01		0.71 ± 0.05		0.063
SAAs ^2^	0.88 ± 0.02		1.62 ± 0.06		0.0004
AAAs ^3^	1.01 ± 0.02		1.28 ± 0.01		0.0003
In vitro DIAAS ^4^	69.6 (Val)		70.9 (Trp)		0.816
(**b**)					
	**v2food^®^ Mince**	**v2food^®^** **Formulation 1**	**v2food^®^** **Formulation 2**	**v2food^®^** **Formulation 3**	***p*-Value ^1^**
DIAA reference ratio					
Threonine	1.48 ± 0.04	1.33 ± 0.03	1.38 ± 0.01	1.37 ± 0.06	0.107
Leucine	1.04 ± 0.02 ^b^	0.91 ± 0.07 ^a^	1.04 ± 0.09 ^b^	0.99 ± 0.02 ^b^	0.0003
Isoleucine	1.03 ± 0.02 ^b^	0.85 ± 0.03 ^a^	1.04 ± 0.02 ^b^	1.03 ± 0.02 ^b^	0.0002
Valine	0.73 ± 0.09 ^b^	0.63 ± 0.02 ^a^	0.74 ± 0.01 ^b^	0.75 ± 0.03 ^b^	0.002
Histidine	2.78 ± 0.17	2.27 ± 0.11	2.27 ± 0.05	2.44 ± 0.12	0.053
Lysine	1.18 ± 0.05	1.03 ± 0.04	1.06 ± 0.01	1.10 ± 0.04	0.095
Tryptophan	0.71 ± 0.05	0.70 ± 0.05	0.83 ± 0.04	0.81 ± 0.02	0.132
SAAs	1.62 ± 0.06 ^a^	2.26 ± 0.08 ^b^	2.15 ± 0.07 ^b^	2.28 ± 0.08 ^b^	0.0003
AAAs	1.28 ± 0.01	1.18 ± 0.01	1.26 ± 0.02	1.22 ± 0.04	0.055
In vitro DIAAS ^4^	70.9 (Trp)	63.0 (Val)	73.9 (Val)	75.3 (Val)	0.072

^1^ Different superscripts denote significant differences between groups, ^2^ SAAs, sulphur amino acids (sum of methionine and cysteine); ^3^ AAAs, aromatic amino acids (sum of phenylalanine and tyrosine); ^4^ Digestible indispensable amino acid score.

**Table 4:** Concentrations and total SCFA concentrations in the in vitro fermentation system following 24 h of in vitro fermentation of digested v2food^®^ mince and control substrates ^1^.

	Control	Cellulose	Inulin	v2food^®^ Mince
Acetic Acid	15.0 ± 0.2 ^a^	15.0 ± 0.3 ^a^	54.5 ± 1.8 ^b^	54.0 ± 4.0 ^b^
Propionic Acid	4.3 ± 0.1 ^a^	4.9 ± 0.1 ^a^	6.8 ± 0.1 ^b^	21.0 ± 0.4 ^c^
Butyric Acid	4.7 ± 0.1 ^a^	4.7 ± 0.1 ^a^	7.4 ± 0.1 ^b^	18.0 ± 1.0 ^c^

^1^ Data are presented as the mean ± SEM. Different superscripts denote significant differences between groups (*p* < 0.05, ANOVA followed by Tukey HSD post hoc analysis).

**Table 5:** The relative abundance (% of total reads—16S rRNA gene sequencing) of bacterial phyla detected in media following 24 h of in vitro fermentation of digested v2food^®^ mince and control substrates ^1^.

Phylum	Control	Cellulose	Inulin	v2food^®^ Mince
Actinomycetota	1.54 ± 0.06 ^a^	1.61 ± 0.19 ^ab^	1.49 ± 0.19 ^a^	2.30 ± 0.18 ^b^
Bacteroidota	10.88 ± 0.62 ^a^	8.40 ± 0.28 ^a^	56.02 ± 0.57 ^b^	11.10 ± 0.95 ^a^
Bacillota	84.32 ± 0.27 ^a^	89.08 ± 0.24 ^b^	42.34 ± 0.47 ^c^	84.63 ± 1.19 ^a^
Lentisphaerota	0.065 ± 0.007 ^a^	0.015 ± 0.008 ^b^	0 ^b^	0.002 ± 0.002 ^b^
Pseudomonadota	3.05 ± 0.52 ^a^	0.76 ± 0.21 ^b^	0.13 ± 0.01 ^b^	1.96 ± 0.59 ^a^
Synergistota	0.017 ± 0.008 ^ab^	0.024 ± 0.006 ^a^	0 ^b^	0 ^b^
Mycoplasmatota	0.078 ± 0.012 ^a^	0.074 ± 0.010 ^a^	0.002 ± 0.002 ^b^	0.004 ± 0.002 ^b^
Verrucomicrobiota	0.019 ± 0.004 ^a^	0.011 ± 0.006 ^ab^	0 ^b^	0.002 ± 0.002 ^b^

^1^ Data are presented as the mean ± SEM. Different superscripts denote significant differences between groups (*p* < 0.05, ANOVA followed by Tukey HSD post hoc analysis).

**Table 6:** The relative abundance (% of total reads—16S rRNA gene sequencing) of bacterial genera detected in media following 24 h of in vitro fermentation of digested v2food^®^ mince or control substrates ^1^.

Genus	Control	Cellulose	Inulin	v2food^®^ Mince
*Alistipes*	0.398 ± 0.028 ^a^	0.210 ± 0.037 ^b^	0.289 ± 0.039 ^ab^	0.063 ± 0.011 ^c^
*Anaerostipes*	0.179 ± 0.008 ^a^	0.138 ± 0.005 ^b^	0.013 ± 0.013 ^c^	0 ^c^
*Bacteroides*	4.30 ± 0.24 ^a^	2.57 ± 0.30 ^b^	1.90 ± 0.07 ^b^	2.40 ± 0.30 ^b^
*Blautia*	9.96 ± 0.18 ^a^	10.48 ± 0.54 ^ab^	3.85 ± 0.31 ^c^	11.76 ± 0.29 ^b^
*Butyricicoccus*	0.766 ± 0.068 ^a^	0.818 ± 0.058 ^a^	0.060 ± 0.013 ^b^	0.758 ± 0.096 ^a^
*Catenibacterium*	0.84 ± 0.12 ^a^	1.13 ± 0.06 ^a^	10.03 ± 1.01 ^b^	0.44 ± 0.08 ^a^
*Clostridium* (family *Clostridiaceae*)	0.389 ± 0.003 ^a^	0.427 ± 0.019 ^a^	0.091 ± 0.022 ^b^	0.306 ± 0.022 ^c^
*Clostridium* (family *Lachnospiraceae)*	2.19 ± 0.16 ^a^	2.14 ± 0.13 ^a^	0.19 ± 0.02 ^b^	1.65 ± 0.20 ^a^
*Collinsella*	1.12 ± 0.04 ^a^	1.12 ± 0.19 ^a^	1.40 ± 0.17 ^ab^	2.01 ± 0.16 ^b^
*Coprococcus*	4.31 ± 0.37 ^a^	4.29 ± 0.27 ^a^	2.33 ± 0.28 ^b^	3.53 ± 0.14 ^ab^
*Dorea*	9.97 ± 0.31 ^a^	12.52 ± 0.81 ^b^	0.45 ± 0.02 ^c^	11.92 ± 0.54 ^ab^
*Eggerthella*	0.109 ± 0.015 ^a^	0.118 ± 0.008 ^a^	0 ^b^	0.053 ± 0.004 ^c^
*Enterobacter*	0 ^a^	0 ^a^	0 ^a^	0.108 ± 0.108 ^a^
*Faecalibacterium*	8.56 ± 0.33 ^a^	6.86 ± 0.06 ^b^	5.76 ± 0.21 ^c^	11.37 ± 0.25 ^d^
*Haemophilus*	0.079 ± 0.013 ^a^	0.43 ± 0.009 ^b^	0 ^c^	0 ^c^
*Klebsiella*	0.007 ± 0.007 ^a^	0 ^a^	0 ^a^	0.141 ± 0.132 ^a^
*Lachnobacterium*	0.015 ± 0.015 ^a^	0.067 ± 0.040 ^ab^	0.170 ± 0.022 ^b^	0 ^ac^
*Lachnospira*	0.519 ± 0.057 ^a^	0.412 ± 0.033 ^ab^	0.043 ± 0.007 ^c^	0.298 ± 0.030 ^b^
*02d06*	0.508 ± 0.057 ^a^	0.423 ± 0.028 ^a^	0.191 ± 0.015 ^b^	0.259 ± 0.022 ^b^
*Odoribacter*	0.113 ± 0.012 ^a^	0.011 ± 0.011 ^b^	0.061 ± 0.009 ^c^	0 ^b^
*Oscillospira*	3.62 ± 0.09 ^a^	4.14 ± 0.22 ^a^	0.37 ± 0.06 ^b^	1.58 ± 0.10 ^c^
*Parabacteroides*	2.42 ± 0.29 ^a^	1.18 ± 0.06 ^b^	0.22 ± 0.01 ^c^	0.57 ± 0.02 ^bc^
*Phascolarctobacterium*	2.27 ± 0.18 ^a^	1.97 ± 0.15 ^a^	0.26 ± 0.02 ^b^	2.31 ± 0.14 ^a^
*Prevotella*	2.93 ± 0.18 ^a^	4.20 ± 0.13 ^a^	53.27 ± 0.68 ^b^	8.02 ± 0.86 ^c^
*Roseburia*	1.47 ± 0.08 ^ab^	2.06 ± 0.11 ^a^	1.01 ± 0.18 ^b^	2.15 ± 0.26 ^a^
*Ruminococcus* (family *Lachnospiraceae*)	3.33 ± 0.18 ^a^	2.77 ± 0.26 ^a^	2.68 ± 0.13 ^a^	3.02 ± 0.37 ^a^
*Ruminococcus* (family *Ruminococcaceae*)	3.01 ± 0.09 ^ac^	3.36 ± 0.13 ^a^	1.09 ± 0.16 ^b^	2.32 ± 0.22 ^c^
*Shigella*	0.019 ± 0.011 ^a^	0.039 ± 0.039 ^a^	0 ^a^	0.125 ± 0.125 ^a^
*Slackia*	0.099 ± 0.007 ^a^	0.133 ± 0.014 ^a^	0.031 ± 0.003 ^b^	0.135 ± 0.009 ^a^
*SMB53*	0.125 ± 0.010 ^ab^	0.136 ± 0.003 ^a^	0.080 ± 0.012 ^bc^	0.067 ± 0.017 ^c^
*Streptococcus*	0.327 ± 0.029 ^a^	0.347 ± 0.030 ^a^	0.054 ± 0.002 ^b^	0.130 ± 0.017 ^b^
*Subdoligranulum*	2.28 ± 0.03 ^a^	2.54 ± 0.09 ^a^	0.50 ± 0.10 ^b^	1.62 ± 0.13 ^c^
*Sutterella*	2.92 ± 0.52 ^a^	0.61 ± 0.15 ^bc^	0.13 ± 0.01 ^b^	1.48 ± 0.12 ^c^

^1^ Data are presented as the mean ± SEM. Different superscripts denote significant differences between groups (*p* < 0.05, ANOVA followed by Tukey HSD post hoc analysis).

**Table 7:** Cellular ferritin production, expressed as mean percentage (±SE) relative to 25 µM FeSO_4_ control, in response to v2food mince formulated with different commercial iron sources without and with 2:1 or 4:1 molar ratios of ascorbic acid.

Cellular Ferritin Production (%)			
Iron Source	Elemental Iron (mg/100 g)		
	2 mg	2.7 mg	4.0 mg
**Iron without ascorbic acid**			
Ferric pyrophosphate	28.0 ± 2.7	35.9 ± 4.1	46.9 ± 2.8
Ferric EDTA	35.0 ± 8.6	32.7 ± 5.3	37.1 ± 7.2
Ferrous sulphate	66.2 ± 6.1 *	86.3 ± 5.8 *	80.6 ± 12.1 *
Ferrous fumarate	47.3 ± 5.8	41.1 ± 6.3	37.5 ± 7.5
Ferrous bisglycinate	49.7 ± 12.4	25.9 ± 3.0	43.7 ± 6.1
Ferrous bisglycinate ± citric acid l	39.0 ± 3.0	53.9 ± 5.1 *	52.7 ± 3.7
**Iron and ascorbic acid (1:2)**			
Ferric pyrophosphate	35.4 ± 1.7 ^#^	45.8 ± 3.0	39.8 ± 2.4
Ferric EDTA	21.0 ± 6.4	36.2 ± 4.1	22.7 ± 2.1
Ferrous sulphate	64.8 ± 8.8 *	66.6 ± 3.5 *	-
Ferrous fumarate	41.9 ± 3.9	63.5 ± 16.5 ^#^	43.6 ± 10.6
Ferrous bisglycinate	31.6 ± 7.0	-	37.9 ± 9.4
Ferrous bisglycinate ± citric acid l	44.0 ± 2.7	46.1 ± 4.9	78.5 ± 5.7 *^#^
**Iron and ascobic acid (1:4)**			
Ferric pyrophosphate	48.8 ± 3.3 ^#^	22.6 ± 4.1	46.4 ± 1.2
Ferric EDTA	31.0 ± 3.9	26.1 ± 4.2	39.3 ± 3.3
Ferrous sulphate	63.9 ± 4.2 *	41.4 ± 7.4	55.2 ± 7.3
Ferrous fumarate	37.5 ± 13.9	25.7 ± 6.3	31.4 ± 5.1
Ferrous bisglycinate	38.0 ± 2.3	20.5 ± 4.4	26.1 ± 3.3
Ferrous bisglycinate ± citric acid l	37.8 ± 3.8	58.8 ± 9.3 *	51.9 ± 6.5
**Lean beef**	298.4 ± 96.3		

* Denotes significant increase (*p* < 0.05) within column groups when compared to ferric pyrophosphate using unpaired *t* tests. ^#^ Denotes significant increase (*p* < 0.05) with ascorbic acid addition using unpaired *t* tests.

## Data Availability

Data are not publicly available due to contractual reasons but can be made available on request.
